# Two New Aristolochic Acid Analogues from the Roots of *Aristolochia* *contorta* with Significant Cytotoxic Activity

**DOI:** 10.3390/molecules26010044

**Published:** 2020-12-23

**Authors:** Hong-Jian Ji, Jia-Yuan Li, Shi-Fei Wu, Wen-Yong Wu, Chang-Liang Yao, Shuai Yao, Jian-Qing Zhang, De-An Guo

**Affiliations:** 1 School of Chinese Materia Medica, Nanjing University of Chinese Medicine, Xianlin Road #138, Nanjing 210023, China; hongjianji2006@163.com (H.-J.J.); wuwenyong1637@163.com (W.-Y.W.); 2 Shanghai Institue of Materia Medica, Chinese Academy of Sciences, Shanghai 201203, China; lijiayuan@simm.ac.cn (J.-Y.L.); biowsf@163.com (S.-F.W.); cpuyao@126.com (C.-L.Y.); yaoshuai@simm.ac.cn (S.Y.); zhangjianqing@simm.ac.cn (J.-Q.Z.); 3 Department of Pharmacy, Yancheng School of Clinical Medicine of Nanjing Medical University, Yancheng 224001, China

**Keywords:** *Aristolochia contorta*, aristolochic acid, cytotoxicity, molecular docking

## Abstract

Twelve compounds, including two new aristolochic acid analogues with a formyloxy moiety (9–10) and 10 known aristolochic acid derivates (1–8 and 11–12), were obtained from the roots of *Aristolochia* *contorta*. Their structures were elucidated using extensive spectroscopic methods. Their cytotoxic activity in human proximal tubular cells HK-2 was evaluated by the MTT method, which has been widely used to assess cell viability. Among these molecules, compounds **3** and **9** were found to be more cytotoxic. Furthermore, molecular modeling was used to evaluate, for the first time, the interactions of compounds **3** and **9** with the target protein organic anionic transporter 1 (OAT1) that plays a key role in mediating aristolochic acid nephropathy. Structure–activity relationships are briefly discussed.

## 1. Introduction

*Aristolochia contorta* is widely distributed in tropical and subtropical regions, especially in the north of China, Korea, and Japan [1]. Previous phytochemical investigations of *A. contorta* revealed that it contains various chemical constituents with noteworthy biological activity, including alkaloids, aristolochic acid analogues, flavonoids, and terpenoids [2,3,4,5,6]. Recently, it was concluded that aristolochic acid (AA) analogues could induce serious nephropathy and cancers based on the results of a significant number of investigations [7,8,9,10], a finding that has aroused wide public concern. Various studies have revealed several mechanisms of aristolochic acid nephropathy. Aristolochic acid analogues with nitro groups at position 10 could be metabolized and activated by hepatic enzymes, which promotes the occurrence of aristolochic acid nephropathy [11]. Specific organic anion transporters mechanism of aristolochic acid derivatives into renal cells may greatly contribute to AA-mediated nephrotoxicity [12]. Other mechanisms, involving SMAD 2/3 and JNK/ERK pathways, mitochondrial/caspase apoptotic pathways, oxidative stress, microRNAs and mRNAs, might contribute to nephrotoxicity [13,14,15]. The progressive lesions and genotoxicity initiated by aristolochic acids are irreversible, and no effective therapeutic treatment for nephrotoxicity has been established up to now.

Due to the irreversible nephrotoxicity of aristolochic acid analogues, *A. contorta* was officially prohibited in the 2020 edition of the Chinese Pharmacopoeia. It is still used as an agricultural product and antitussive in addition to the treatment of snakebites and stomach aches, administered by a doctor or, occasionally, in self-treatment [16].

**Figure 1:**
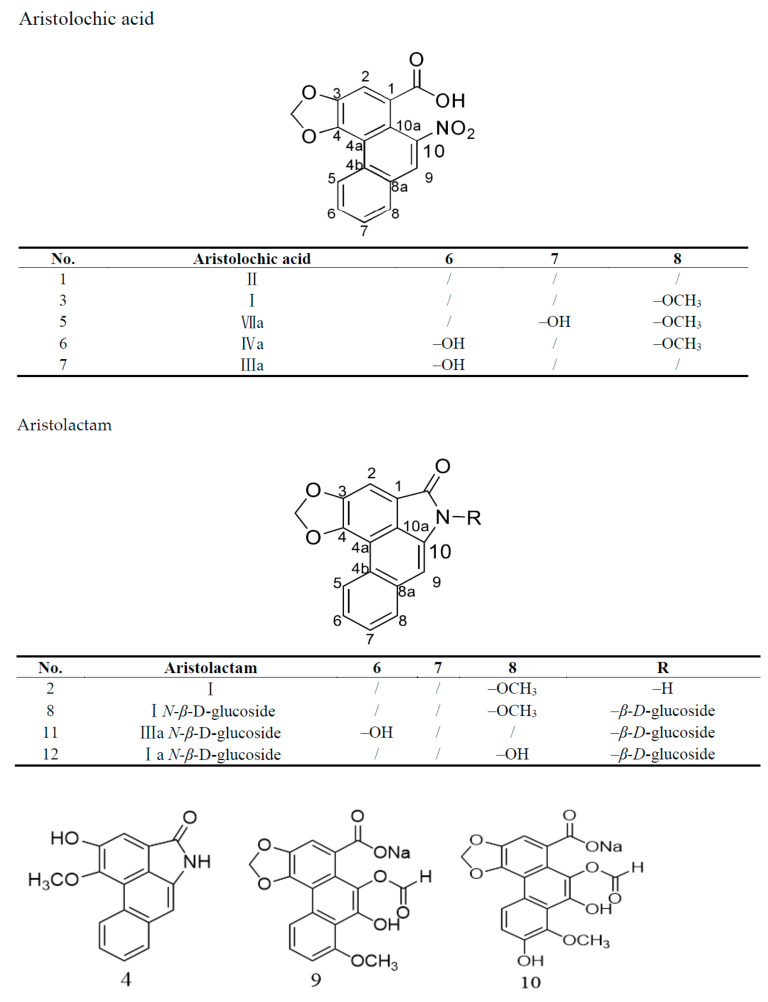
Chemical structures of compounds **1**–**12**.

Motivated by the diverse structures and special bioactivity of aristolochic acid analogues, a phytochemical study on *A. contorta* was conducted. This led to the isolation of two new aristolochic acid analogues (9 and 10) with a formyloxy group at position C10, together with 10 known aristolochic acid derivatives (1–8 and 11–12, Figure 1).

Their structures were determined by extensive spectroscopic analysis, including UV, IR, HR-ESI–MS, 1D and 2D NMR (^1^H, ^13^C, heteronuclear multiple bond correlation spectroscopy (HSQC), heteronuclear single quantum correlation spectroscopy (HMBC)), and inductively coupled plasma mass spectrometry (ICP–MS). The cytotoxicity of most of these compounds against HK-2 cells was assayed, and the interaction of the two new compounds with a target protein was studied by molecular docking.

## 2. Results and Discussion

Compound **9** was collected as a yellowish powder. Its molecular formula, C_18_H_11_O_8_Na, was established by HR-ESI–MS spectrometry, according to its adduct ions [M + H]^+^ (C_18_H_12_O_8_Na) at *m*/*z* 379.0341, [M + H_2_O + H]^+^ (C_18_H_14_O_9_Na) at *m*/*z* 397.0461, and [M + H_2_O − H]^−^ (C_18_H_12_O_9_Na) at *m*/*z* 395.0348, indicating that its molecular weight is 378.

UV absorption occurred at 258, 286 (sh), 314 (sh), and 390 nm. Furthermore, the IR spectrum absorption band at 749 cm^−1^ showed the presence of a typical phenanthrene skeleton [17].

**Figure 2:**
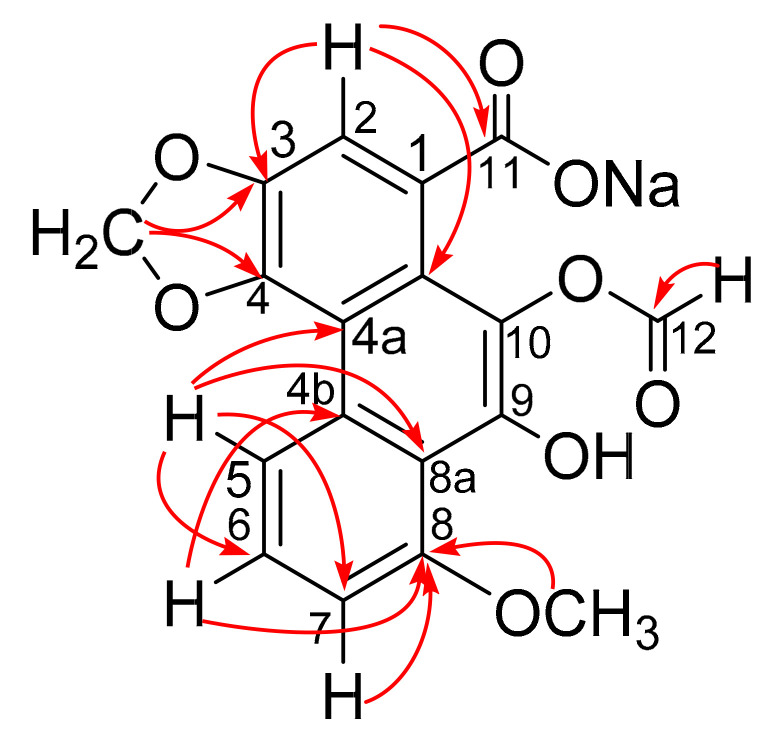
HMBC correlation (H→C) for compound **9**.

The ^1^H NMR and ^13^C NMR data of compound **9** showed signals indicating the presence of a formyl group at δ_H_ 8.22 (1 H, s, –CHO), δ_C_ 161.5 (–CHO), an ABX system aromatic ring at δ_H_ 8.78 (1 H, d, *J* = 8.3 Hz, H-5), δ_C_ 119.6 (C-5), δ_H_ 7.76 (1 H, t, *J* = 8.3 Hz, H-6), δ_C_ 128.4 (C-6) and δ_H_ 7.44 (1 H, m, H-7), *δ*_C_ 109.0 (C-7), an aromatic proton singlet at *δ*_H_ 7.45 (1H, s, H-2), *δ*_C_ 108.5 (C-2), a typical methylenedioxy group at *δ*_H_ 6.42 (2H, s), *δ*_C_ 102.1 (–OCH_2_O–), and a methoxy group at *δ*_H_ 4.18 (3H, s, –OCH_3_), *δ*_C_ 56.3 (–OCH_3_), based on the HSQC spectrum. All the above-mentioned signals demonstrate that compound **9** is an aristolochic acid analogue that shares the same skeleton as aristolochic acid I. The comparison of the molecular weight and 1D NMR data of aristolochic acid I and compound **9** indicated that the nitro group was missing in the latter. Meanwhile, a hydroxy group and a formyl moiety were observed. The formyloxy group was located at C-10, which was confirmed by the HMBC correlations from H-12 to C-10 (Figure 2).

The cation portion was identified as a sodium ion by ICP–MS.

Consequently, compound **9** was established as sodium 9-hydroxy-10-formyloxy aristolochate I. The HR-ESI–MS, UV, and 1D and 2D NMR spectra for compound **9** are shown in Figures S1–S7. This is the first report of a formyloxy derivative of aristolic acid, which is confirmed to be a natural rather than an artificial product, isolated by LC–MS from the methanol extract of *A. contorta*. (Figure S8). ^1^H and ^13^C NMR spectral data are shown in Table 1.

Compound **10** was obtained as a yellowish powder. The molecular formula was established as C_18_H_11_O_9_Na by HR-ESI–MS spectrometry, which gave ions at [M + H]^+^ (C_18_H_12_O_9_Na) at *m*/*z* 395.0379, [M + H_2_O + H]^+^ (C_18_H_14_O_10_Na) at *m*/*z* 413.0485, and [M + H_2_O − H]^−^ (C_18_H_12_O_10_Na) at *m*/*z* 411.0286, indicating its molecular weight to be 394. The molecular weight of compound **10** was 16 mass units higher than that of compound **9**, which is consistent with the presence of a hydroxyl group in compound **10**.

The UV spectrum showed absorption at 248, 290(sh), 328(sh), and 398 nm, similar to what observed for compound **9**.

The ^1^H NMR data revealed a formyl group at *δ*_H_ 8.22 (1H, s, –CHO), four aromatic protons signals, including an AB system at *δ*_H_ 7.16, (1H, d, *J* = 8.80 Hz, H-5) and 7.36 (1H, d, *J* = 8.75 Hz, H-6), a singlet at *δ*_H_ 7.41 (1H, s), a typical methylenedioxy group at *δ*_H_ 6.42 (2H, s), and a methoxy group at *δ*_H_ 4.18 (3H, s, –OCH_3_). The ^13^C NMR spectrum of compound **10** showed 18 carbon signals, including 16 *sp*^2^ carbons and two *sp*^3^ carbons.

Ten of the carbon signals at 128.8 (C-1), 108.2 (C-2), 146.1 (C-3), 146.7 (C-4), 116.1 (C-5), 111.9 (C-6), 147.0 (C-7), 149.9 (C-8), 161.9 (C-9), and 144.7 (C-10) were assigned to the phenanthrene skeleton through comparison with the NMR data of compound **9**.

Comparison of the NMR data of compounds **9** and **10** revealed a difference due to the presence of a hydroxyl group at C-7 in the latter, which was confirmed by the HMBC correlations from H-5 (*δ*_H_ 7.16), H-6 (*δ*_H_ 7.36), and 8-OCH_3_ (*δ*_H_ 4.09) to C-7 (*δ*_C_ 147.0).

Consequently, compound **10** was established as sodium 7,9-dihydroxy-10-formyloxy aristolochate I.

The ^1^H NMR and ^13^C NMR spectral data are shown in Table 1. The HR-ESI–MS, UV, and 1D and 2D NMR spectra for compound **10** are available in Figures S9–S13. Compound **10** was also verified to be a natural rather than an artificial product, isolated by LC–MS from the methanol extract of *A. contorta*, as shown in Figure S14.

The other 10 compounds (**1**–**8** and **11**–**12**) were identified as aristolochic acid II [1], aristolactam I [2], aristolochic acid I [3], aristolactam AII [4], aristolochic acid VIIa [5], aristolochic acid IVa [6], aristolochic acid IIIa [7], aristolactam I*N*-β,d-glucoside [8], aristolactam IIIa *N*-β,d-glucoside [11], and aristolactam Ia *N*-β,d-glucoside [12] by comparison with the NMR data present in the literature [18,19].

**Figure 3:**
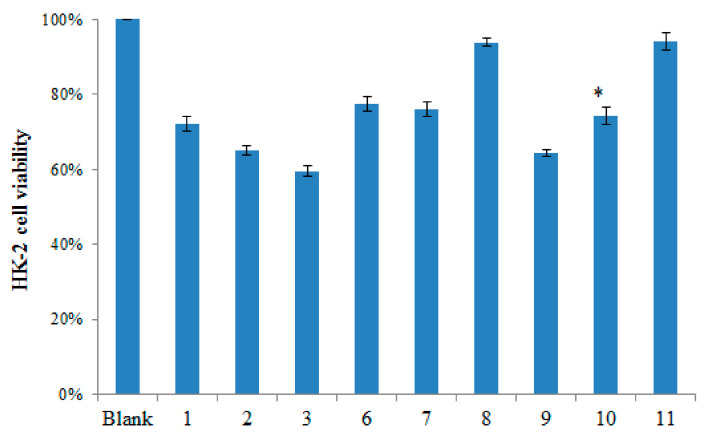
Cytotoxicity of the isolated compounds. HK-2 cells were treated with various aristolochic acid (AA) derivatives for 24 h, and then cell cytotoxicity was detected by the MTT assay. Student’s t-test was used to assess differences between the treatment groups; *p* values < 0.05 were considered statistically significant (* ≤0.05, for compounds **9** and **10** compared with compound **3**).

Based on the nephrotoxicity of aristolochic acid, the cytotoxic activity of compounds **1**–**3** and **6**–**11** was evaluated in HK-2 cells using the 3-(4,5-dimethylthiazol-2-yl)-2,5-diphenyltetrazolium bromide (MTT) colorimetric assay [20]. Untreated cells were used as control. The relative cell viability resulting from the treatment with 20 μmol/L of the different isolated compounds was measured. The results are shown in Figure 3.

AAI and compound **9** showed significant cytotoxic effects in human HK-2 cells, while compounds **1**, **2**, **6**, **7**, and **10** had moderate cytotoxicity, and compounds **8** and **11** showed marginal cytotoxicity in HK-2 cells.

Usually, AAI is transformed to *N*-hydroxyaristolactams (AL-NOHs) by a nitroreduction reaction by the liver enzymes CYPs, especially CYP1A2. Then, it is converted to aristolactam nitrenium, which is an electrophilic cyclic aristolactam nitrenium ion with delocalized positive charges. It preferentially binds to the exocyclic amino groups of purine bases in the DNA to form AA–DNA adducts. These adducts can lead to A→T transversions and thus cause renal disease and cancers. Therefore, a nitro group in position 10 may play a key role in AAI mutagenicity and genotoxicity [11]. Similarly, aristolochic acid analogues with a nitro group located at C10 are mutagenic and genotoxic [21]. Besides, recently an increasing number of investigations have reported that organic anion Transporters (OATs) proteins, located at the basolateral membrane of the proximal tubules, especially OAT1 and OAT3, facilitate the uptake of AAI by renal cells, which at least partly leads to site-selective AAI-induced nephrotoxicity [8]. The results of the cytotoxicity test showed significant apoptosis of HK-2 cells upon treatment with AAI, containing the nitro group at the C10 position,a and with compound **9**, containing the formyloxy group at the C10 position instead of the nitro group. It was also shown that not only the nitro group but also the formyloxy group at C10 plays an important role in AA-mediated cytotoxicity by OATs. The cytotoxicity results for the above compounds suggested a relationship between structure and cytotoxicity involving different functional groups at the C10 position, such as a formyloxy group. 

**Figure 4:**
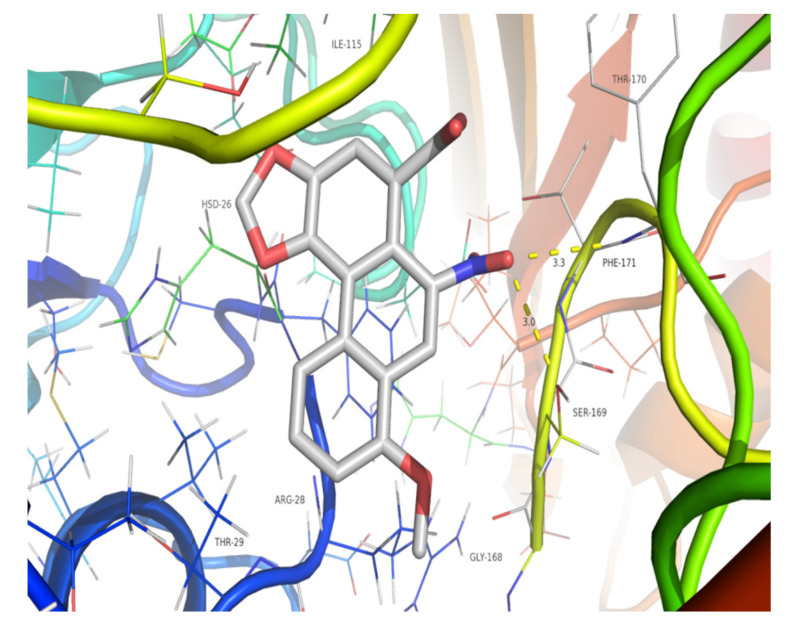
Binding pose of compound **3** docked into the protein 4NOG. The yellow dotted lines indicate hydrogen bonds between compound **3** and 4NOG.

**Figure 5:**
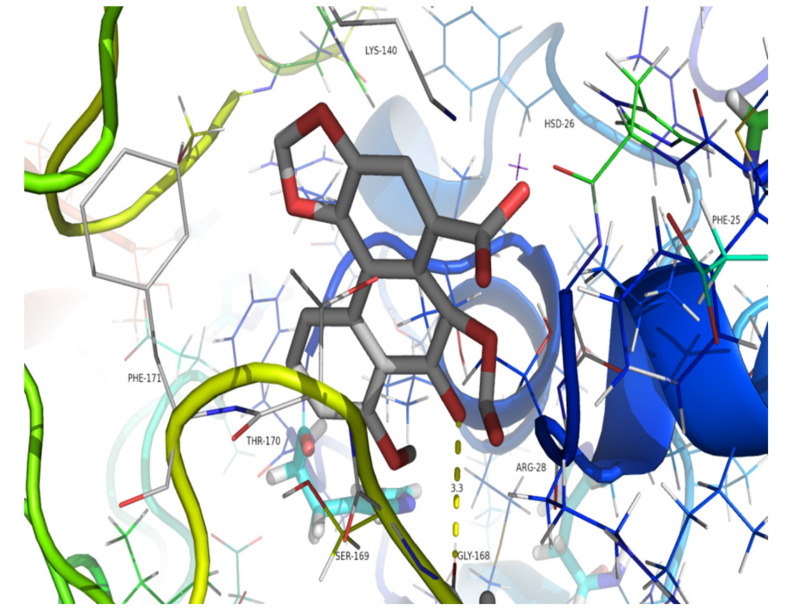
Binding pose of compound **9** docked into the protein 4NOG. The yellow dotted line indicates a hydrogen bond between compound **9** and 4NOG.

Since compound **9** does not have a nitro group at C10, OATs, rather than metabolic activation by liver enzymes, may play an important role in nephrotoxicity by facilitating the uptake of this aristolochic acid analogue into renal cells. OAT1, in particular, plays a key role in nephrotoxicity by facilitating the uptake of aristolochic acid into renal cells [22,23]. Since the structural characterization of OAT1 is lacking, we conducted homology modeling using the available structure of an OAT1 analogue, PDB code 4NOG, and a structure was generated by the value of global model quality estimation (GMQE) based on the SWISS model (Figure S17). Then, molecular docking of compounds **3** and **9** with the generated structure of OAT1 was performed using LeDock software. The average values of the binding free energy calculated for compounds **3** and **9** were −4.34 and −4.23 kcal/mol, respectively. The calculated binding free energies of compounds **3** and **9** were compared, and the *p* value was 0.17, which is greater than 0.05. This shows that the two compounds might interact almost equally with the target protein. The molecular interactions between these compounds and the active sites of the OTA1 analogue 4NOG were analyzed in terms of hydrogen bonding. Compound **3** showed stability due to conventional hydrogen bond interactions with Phe171 (3.3 Å) and Ser169 (3.0 Å) (Figure 4), while compound **9** presented strong hydrogen bond interactions with Gly168 (3.3 Å) (Figure 5). The predicted binding free energy of compound **9** to 4NOG was slightly lower than that of compound **3**, but the distance between compound **9** and Gly27 is shorter by 0.4 Å. Gly27 residue in 4NOG protein may be important to maintain the geometry of the binding gorge and provide electrostatic balance. The theoretical results obtained by molecular docking for compounds **9** and compound **3** were in agreement with the in vitro assays.

Further research is needed to better elucidate the structure–toxicity relationship.

## 3. Materials and Methods

### 3.1. General Information

UV spectra were recorded in methanol using an Agilent 1200 diode array detector (Agilent Co., American). IR spectra were recorded on a NICOLET iS10 FTIR spectrometer on KBr pellets (Thermo Co., American). NMR spectra were recorded on a Bruker AVANCE III 500 spectrometer (chemical shift values are presented as δ values with TMS as the internal standard; Bruker Co., Billerica, MA, USA). HR-ESI–MS data were measured on a Waters Xevo G2 Q-TOF mass spectrometer (Waters Co., Milford, MA, USA). Semi-preparative HPLC was performed on an Agilent 1100 system consisting of a G1312A binary pump with a diode array detector. Preparative HPLC was performed on a Hanbo system consisting of a binary pump with a Nu3000 UV detector. A XBridge prep C18 column (5 μm, 250 mm × 10 mm, made in Ireland), an Agilent ZORBAX SB-C18 column (5 μm, 250 mm × 10 mm, made in USA), and a Hedera ODS-2 C18 column (10 μm, 100 Å, 250 mm × 10.0 mm, made in China) were used for preparation. Column chromatography was performed using silica gel (200–300 mesh, Shanghai Scientific Co., Ltd., Shanghai, China). TLC was conducted on silica gel GF 254 (Merk Chemicals Co., Ltd., Shanghai, China) plates. All solvents used in column chromatography and HPLC were of analytical grade (Sinopharm Chemical Reagent Co., Ltd., Shanghai, China) and chromatographic grade (Sinopharm Chemical Reagent Co., Ltd., Shanghai, China), respectively. The melting points (mps) of aristolochic acid analogues were determined by an SGW X-4 micro melting point apparatus (Shanghai Precision & Scientific Instrument Co., Ltd., Shanghai, China)

Human HK-2 renal proximal convoluted tubule epithelial cells were purchased from the American Type Culture Collection (ATCC, Rockville, MD, USA).

### 3.2. Materials

The roots of *A. contorta* were collected from Bozhou, Anhui Province, China, in November 2018 and authenticated by Professor Shuai Yao from Shanghai Institute of Materia Medica, Chinese Academy of Science.

### 3.3. Extraction and Isolation

Dried and powdered roots of *A. contorta* (5.0 kg) were extracted with methanol (20 L) for 24 h at room temperature, for three times. The solvent was removed in vacuo to obtain a methanol extract (1.1 kg). The methanol extract was mixed with 2 L of distilled water using an ultrasonicator (200 W, 59 kHz, 30 min) and partitioned sequentially with extracted petroleum ether (60–90 °C) (3 × 2 L), ethyl acetate (3 × 2 L), dichloromethane (3 × 2 L), and water-saturated n-butyl alcohol (3 × 2 L). The ethyl acetate phase and dichloromethane phase were combined and concentrated under vacuum (70 g). Then, they were injected into a silica gel column and eluted with various ratios of petroleum ether–ethyl acetate (containing 0.2% formic acid) (40:1, 20:1, 10:1, 1:10, 1:20, and 1:40 *v*/*v*) to yield 18 fractions: Fr. 1 (4.0 g), Fr. 2 (3.6 g), Fr. 3 (4.2 g), Fr. 4 (3.2 g), Fr. 5 (3.0 g), Fr. 6 (2.8 g), Fr. 7 (4.4 g), Fr. 8 (5.2 g), Fr. 9 (3.8 g), Fr. 10 (3.3 g), Fr. 11 (3.3 g), Fr. 12 (3.1 g), Fr. 13 (4.5 g), Fr. 14 (5.0 g), Fr. 15 (3.7 g), Fr. 16 (3.9 g), Fr. 17 (4.8 g), and Fr. 18 (4.2 g). Fr. 2 (3.6 g) was further separated by semi-preparative HPLC (MeOH–H_2_O, 60:40, containing 0.2% formic acid, *v*/*v*), using a Hedera ODS-2 C18 column at 15 mL/min and UV at 254 nm to obtain compounds **2** (32 mg, tR 14 min) and **1** (8 mg, tR 18 min). Fr. 4 (3.2 g) was further separated by semi-preparative HPLC (acetonitrile–H_2_O, 40:60, containing 0.2% formic acid, *v*/*v*), XBridge, and a C18 column at a rate of 4 mL/min and UV at 254 nm to obtain compound **4** (5 mg, tR 13.4 min). Fr. 3 (4.2 g) was used to prepare TLC with silica gel GF 254 (methylbenzene–ethyl acetate, 1:1, containing 0.2% formic acid *v*/*v*) to get four sub-fractions (sub-Fr. 3-1–4). Sub-Fr. 3-1 (326.4 mg) was further separated by semi-preparative HPLC (MeOH–H_2_O, 55:45, containing 0.2% formic acid *v*/*v*) and a ZORBAX SB C18 column at a rate of 3.5 mL/min and UV at 254 nm to yield compound **3** (6.7 mg, tR 12.1 min), and sub-Fr. 3-2 (261.4 mg) was further separated by semi-preparative HPLC (MeOH–H_2_O, 60:40, containing 0.2% formic acid *v*/*v*) ZORBAX SB C18 column, 4.0 mL/min, 254 nm) to yield compound **5** (9.7 mg, tR 9.8 min). Sub-Fr. 3-3 (138 mg) was further purified by semi-preparative HPLC (MeOH–H_2_O, 55:45, containing 0.2% formic acid *v*/*v*), using a ZORBAX SB C18 column at a rate of 4.0 mL/min and UV at 254 nm to yield compound **6** (15.6 mg, tR 10.2 min). Fr. 5 (3.2 g) was further separated by semi-preparative HPLC (acetonitrile–H_2_O, 40:60, containing 0.2% formic acid *v*/*v*) and a Hedera ODS-2 C18 column at a rate of 18 mL/min and UV at 254 nm to obtain compound **7** (32 mg, tR 13 min). Fr. 6 (2.8 g) was further separated by semi-preparative HPLC (acetonitrile–H_2_O, 42:58, containing 0.2% formic acid *v*/*v*, ZORBAX SB C18 column, 4.0 mL/min, UV at 254 nm) to yield compound **8** (4 mg, tR 13.7 min). Fr. 8 (5.2 g) was further separated by semi-preparative HPLC (acetonitrile–H_2_O, 35:65, containing 0.2% formic acid *v*/*v*), XBridge, and a C18 column at a rate of 4 mL/min and UV at 254 nm to obtain compounds **9** (4 mg, tR 10.4 min) and **10** (2.0 mg, tR 16.4 min). Fr. 11 (3.3 g) was further separated by semi-preparative HPLC (MeOH–H_2_O, 40:60, containing 0.2% formic acid *v*/*v*), XBridge, and a C18 column at a rate of 3.6 mL/min and UV at 254 nm to obtain compounds **11** (3.0 mg, tR 11.6min) and **12** (2.0 mg, tR 18.4 min).

#### 3.3.1. Aristolochic Acid II (1)

Yellowish powder with the following characteristics: HR-ESI–MS *m*/*z* 310.0353 [M − H]^−^ (C_16_H_8_NO_6_, calculated 310.0352), mp: 262~263 °C, UV absorbance (methanol): 220, 252, 302, 377 nm. ^1^H NMR and ^13^C NMR spectral data are shown in Table S15.

#### 3.3.2. Aristolactam I (2) 

Yellowish powder with the following characteristics: HR-ESI–MS *m*/*z* 294.0769 [M + H]^+^ (C_17_H_12_NO_4_, calculated 294.0766), mp > 300 °C, UV absorbance (methanol): 241, 260, 296, 335, 396 nm. ^1^H NMR and ^13^C NMR spectral data are shown in Table S16.

#### 3.3.3. Aristolochic Acid I (3)

Yellow powder with the following characteristics: HR-ESI–MS *m*/*z* 359.0879 [M + NH_4_]^+^ (C_17_H_15_N_2_O_7_, calculated 294.0874), mp: 283~285 °C, UV absorbance (methanol): 225, 253(sh), 321, 394 nm. ^1^H NMR and ^13^C NMR spectral data are shown in Table S15.

#### 3.3.4. Aristolactam AII (4)

Yellowish powder with the following characteristics: HR-ESI–MS *m*/*z* 266.0821[M + H]^+^ (C_16_H_12_NO_3_, calculated 266.0817), mp: 271~272 °C, UV absorbance (methanol): 234, 276, 286, 390 nm. ^1^H NMR and ^13^C NMR spectral data are shown in Table S16.

#### 3.3.5. Aristolochic Acid VIIa (5)

Orange powder with the following characteristics: HR-ESI–MS *m*/*z* 356.0408 [M − H]^−^ (C_17_H_10_NO_8_, calculated 356.0406), mp > 300 °C, UV absorbance (methanol): 228, 270, 316 (sh), 385 nm. ^1^H NMR and ^13^C NMR spectral data are shown in Table S15.

#### 3.3.6. Aristolochic Acid IVa (6)

Orange powder with the following characteristics: HR-ESI–MS *m*/*z* 356.0408 [M − H]^−^ (C_17_H_10_NO_8_, calculated 356.0406), mp: 261~262 °C, UV absorbance (methanol): 225, 244, 256, 283 (sh), 318, 397 nm. ^1^H NMR and ^13^C NMR spectral data are shown in Table S16.

#### 3.3.7. Aristolochic Acid IIIa (7)

Yellowish powder with the following characteristics: HR-ESI–MS *m*/*z* 326.0292 [M − H]^−^ (C_16_H_8_NO_7_, calculated 326.0301), mp: 278~229 °C, UV absorbance (methanol): 215, 257, 308 nm. ^1^H NMR and ^13^C NMR spectral data are shown in Table S15.

#### 3.3.8. Aristolactam I *N*-β,D-Glucoside (8)

Yellowish powder with the following characteristics: HR-ESI–MS *m*/*z* 456.1296 [M + H]^+^ (C_23_H_22_NO_9_, calculated 456.1295), mp > 300 °C, UV absorbance (methanol): 214, 241, 255, 297, 355, 394 nm. ^1^H NMR and ^13^C NMR spectral data are shown in Table S16.

#### 3.3.9. Aristolactam IIIa *N*-β,D-glucoside (11)

Yellowish powder with the following characteristics: HR-ESI–MS *m*/*z* 442.1143 [M + H]^+^ (C_22_H_20_NO_9_, calculated 442.1138), mp > 300 °C, UV absorbance (methanol): 212, 236, 266, 283, 350, 366, 398 nm. ^1^H NMR and ^13^C NMR spectral data are shown in Table S16.

#### 3.3.10. Aristolactam Ia *N*-β,D-Glucoside (12)

Yellowish powder with the following characteristics: HR-ESI–MS *m*/*z* 440.00971 [M − H]^−^ (C_16_H_8_NO_7_, calculated 440.0982), mp > 300 °C, UV absorbance (methanol): 240, 260, 298, 332, 397 nm. ^1^H NMR and ^13^C NMR spectral data are shown in Table S16.

### 3.4. MTT Assay

The MTT assay is a colorimetric assay for measuring cell metabolic activity. HK-2 cells were purchased from Shanghai Institutes for Biological Sciences, Chinese Academy of Science, and cultured in high-glucose Dulbecco’s modified Eagle’s medium (DMEM) containing 10% (*v*/*v*) fetal bovine serum (FBS, Gibco, USA) and 1% penicillin–streptomycin at 37 °C in a humidified atmosphere with 5% CO_2_. The cells were seeded in 96-well culture plates (2 × 10^4^ cells/well) and treated with 100 μL of different concentrations of the target compounds for 24 h. After 24 h of treatment, 20 μL of MTT solution was added, and the cells were further incubated for an additional 4 h. Formazan precipitates were dissolved with dimethyl sulfoxide (150 μL/well), and then, the absorbance was measured at 490 nm with a microplate reader (Bio-Rad model 680, O-524). 

### 3.5. Molecular Modeling

Docking was performed using LeDock software [24]. Although organic anionic transporter 1 (OAT1) plays a key role in aristolochic acid-induced nephropathy, the three-dimensional structure of OAT1 was not available. Homology modeling was conducted using SWISS MODEL to solve the dilemma [25]. The target sequence of OAT1 appeared to be as follows: ARKWGYEKKKIPKDEAIIVSCCGCFHGRTLGVISMSCDNEATRGFGPLLPGHVKVDFGDEVALEKIFKEKGDRIAGFLFEPIQGEAGVIIPPDGYLKAIRDLCTKYNILMVADEIQTGRTGRMLACDWEEIRPDVVILGKALGGGVVPVSAVLADKDVMLCIQPGEHGSTFGGNPMASAVAVASLEVIKDEKLAERSDQMGQELRHQLIKVQQQFPNVIKEVRGKGLFNAVELNSKALFPVSAYDICIKLKERGILAKPTHDTIIRLTPPLCISLEELQGGSKALQDVLELDLPKMQMQNAKPETLPSTASHVCDRCGRNS [26]. Based on the above sequence, an OAT1 protein model was generated by the homology modeling server. Compared with templates of proteins in the database, ornithine aminotransferase (PDB code: 4NOG) with a ligand had the highest value of GMQE [27]. According to Xiang [28], a protein sequence with over 30% identity to a known structure can often be predicted with an accuracy equivalent to that of a low-resolution X-ray structure; 4NOG generated with ligand by SWISS MODEL software revealed 45.39% identity with OAT1 (Figure S18). Therefore, 4NOG instead of OAT1 was considered appropriate for molecular docking.

The binding poses of AAI and compound **9** docked into 4NOG are shown in Figure 4 and Figure 5, respectively. The parameters of molecular docking were as follows: *X* min: 19.367, *X* max: 34.538; *Y* min: −4.364, *Y* max: 9.336; *Z* min: 51.478, *Z* max: 65.026. Docking of compound **3** or **9** with the target protein was performed 10 times by LeDock software. Then, the binding free energy values were calculated using independent samples T Tests with SPSS 22.0. 

## 4. Conclusions

In this study, two new aristolochic acid analogues (**9** and **10**), together with 10 known compounds (**1**–**8** and **11**–**12**), were isolated from the roots of *A. contorta*. The toxicity of most of the isolated compounds was evaluated based on their ability to induce apoptosis in HK-2 cells. The newly obtained compound **9** and the known compound **3** showed significant cytotoxicity in HK-2 cells, which was supported by the molecular modeling results. The results of this study not only enrich our knowledge of the structures of the compounds obtained from *A. contorta* but also expand our understanding of their structure–toxicity relationships.

## Figures and Tables

**Table 1:** ^1^H (500 MHz) and ^13^C NMR data (125 MHz) of compounds **9** and **10** (in DMSO-*d*_6_, δ in ppm, *J* in Hz).

	9			10	
C	δ_H_	δ_C_	C	δ_H_	δ_C_
1		128.0	1		128.8
2	7.45, s	108.5	2	7.41, s	108.3
3		143.9	3		146.1
4		146.1	4		146.7
4a		117.1	4a		115.5
4b		128.0	4b		128.8
5	8.78, d (8.3)	119.6	5	7.16, d (8.80)	116.1
6	7.77, t (8.3)	128.4	6	7.36, d (8.75)	111.9
7	7.44, d (6.8)	109.0	7		147.0
8		153.9	8		149.9
8a		115.3	8a		119.8
9		161.5	9		161.9
10		145.8	10		144.7
10a		118.6	10a		118.6
–OCH2O–	6.42, s	102.1	–OCH2O–	6.27, s	101.6
CH3O–	4.18, s	56.3	CH3O–	4.09, s	57.1
–OCHO	8.22, s	161.5	–OCHO	8.20, s	162.0
–COONa		171.6	–COONa		172.0

## Data Availability

All data and models generated or used during the study appear in the submitted article.

## Supplementary Materials

Supplementary materials are available online. Figures S1–S8: HR-ESI–MS, UV, IR, 1D and 2D NMR spectra and LC–MS analysis of compound **9** and methanol extract from root of *A. contorta* ; Figures S9–S14: HR-ESI–MS, UV, 1D and 2D NMR, and HR-ESI–MS data for compound **10** and methanol extract from root of *A. contorta*; Tables S15–S16: 1H NMR (DMSO-d6, 500 MHz) and 13C NMR (DMSO-d6, 125 MHz) spectral data for compounds **1**–**8** and **11**–**12**; Figure S17: structure of PDB code 4NOG; Figure S18: 4NOG generated with ligand by SWISS-MODEL software.

## Sample Availability

Samples of the compounds **1**–**12** are available from the authors.
