# Supplementary material for: Two New Aristolochic Acid Analogues from the Roots of Aristolochia contorta with Significant Cytotoxic Activity
Source: Molecules. 2020 Dec 23;26(1):44. doi: 10.3390/molecules26010044 (PMC7795626; doi:10.3390/molecules26010044)
Supplement: Supplementary file 1 [file molecules-26-00044-s001.pdf]

Two New Aristolochic Acid Analogues from the Roots of *Aristolochia contorta* with Significant Cytotoxic Activity

Hong-Jian Ji [1,2,3†], Jia-Yuan Li [2†], Shi-Fei Wu [2], Chang-Liang Yao [2], Shuai Yao [2], Jian-Qing Zhang [2] and De-An Guo [1,2,*]

1.School of Chinese Materia Medica, Nanjing University of Chinese Medicine , Haike Road #501, Shanghai 201203, China; hongjianji2006@163.com (H.-J.J.); lijiayuan@simm.ac.cn (J.-Y.L.); biowsf@163.com (S.-F.W.); cpuyao@126.com (C.-L.Y.); zhangjianqing@simm.ac.cn (J.-Q.Z.); daguo@simm.ac.cn (D.-A.G.)

2.Shanghai Institue of Materia Medica, Chinese Academy of Sciences Shanghai 201203, China

3.Department of Pharmacy, The Yancheng School of Clinical Medicine of Nanjing Medical University, Yancheng 224001, China

*Correspondence: daguo@simm.ac.cn (D.-A.G.);

Tel.: +86-021-50271516 (D.-A.G.)

Supporting Information Content:

S1. The HR-ESI–MS spectrum of compound **9.**

S2. The UV spectrum of compound **9** (methanol).

S3. The IR spectrum of compound **9.**

S4. The ^{1}H NMR spectrum of compound **9** (DMSO-d_6, 500 MHz).

S5. The ^{13}C NMR spectrum of compound **9** (DMSO-d_6, 125 MHz).

S6. The HSQC spectrum of compound **9** (DMSO-d_6, 500 MHz).

S7. The HMBC spectrum of compound **9** (DMSO-d_6, 500 MHz).

S8. A: tR of compound 9;

B: *m/z* of compound 9 at the same retention time;

C: TIC of the methanol extract from root of A.*contorta* by LC-Q-TOF/MS;

D: EIC of *m/z* 397.03 from the methanol extract from root of A.*contorta* by LC-Q-TOF/MS under the same liquid chromatography and mass spectrometry conditions;

E: Compound **9** was identified from the methanol extract from root of A. *contorta* by LC-Q-TOF/MS under the same liquid chromatography and mass spectrometry conditions. As indicated by symbols # and *.

S9.The HR-ESI–MS spectrum of compound **10.**

S10.The UV spectrum of compound **10** (methanol).

S11.The ^{1}H NMR spectrum of compound **10** (DMSO-d_6, 500 MHz).

S12.The ^{13}C NMR spectrum of compound **10** (DMSO-d_6, 125 MHz).

S13.The HSQC spectrum of compound **10** (DMSO-d_6, 500 MHz).

S14. F: tR of compound 10;

G: *m/z* of compound 10 at the same retention time;

H: TIC of the methanol extract from root of A.*contorta* by LC-Q-TOF/MS;

I: EIC of *m/z* 413.035 from the methanol extract from root of A.*contorta* by LC-Q-TOF/MS under the same liquid chromatography and mass spectrometry conditions;

J: Compound **10** was identified from the methanol extract from root of A. *contorta* by LC-Q-TOF/MS under the same liquid chromatography and mass spectrometry conditions. As indicated by symbols & and δ .

S15.^{1}H NMR (DMSO-d_6, 500 MHz) and ^{13}C NMR (DMSO-d_6, 125 MHz) spectral data for compounds **1, 3, 5, 6,** and **7**.

S16.^{1}H NMR (DMSO-d_6, 500 MHz) and ^{13}C NMR (DMSO-d_6, 125 MHz) spectral data for compounds **2, 4, 8, 11,** and **12**.

S17.Structure of PDB code **4NOG**.

S18.**4NOG** generated with ligand by SWISS-MODEL software get 45.39% identity with OAT1.

S1. HR-ESI–MS spectrum of compound **9.**

HR-ESI(+) –MS spectrum of compound **9.**

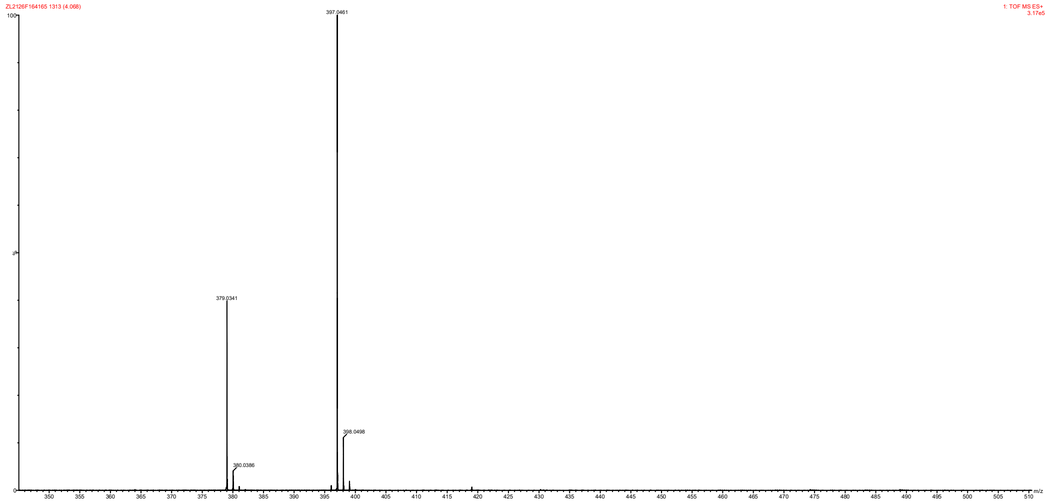

HR-ESI(−) –MS spectrum of compound **9.**

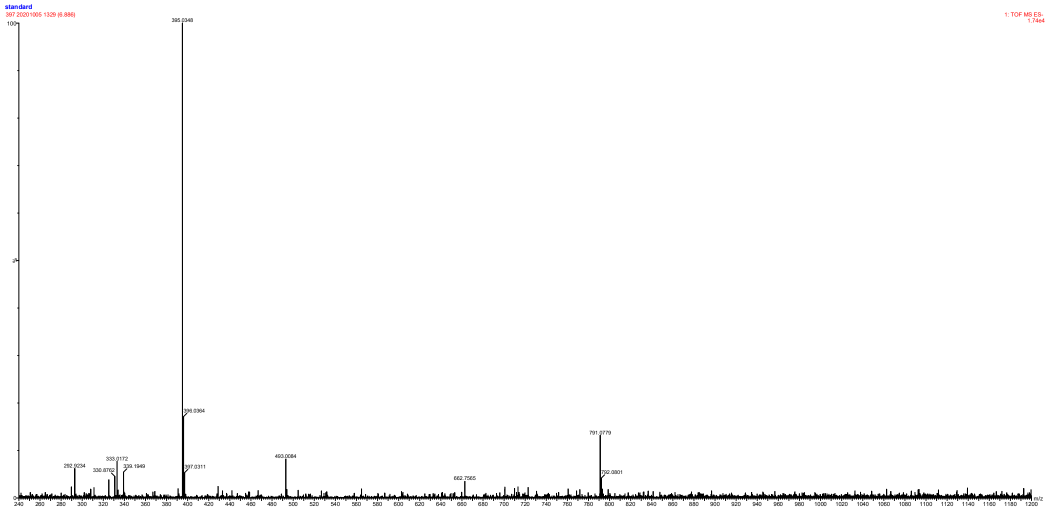

S2. UV spectrum of compound **9** (methanol).

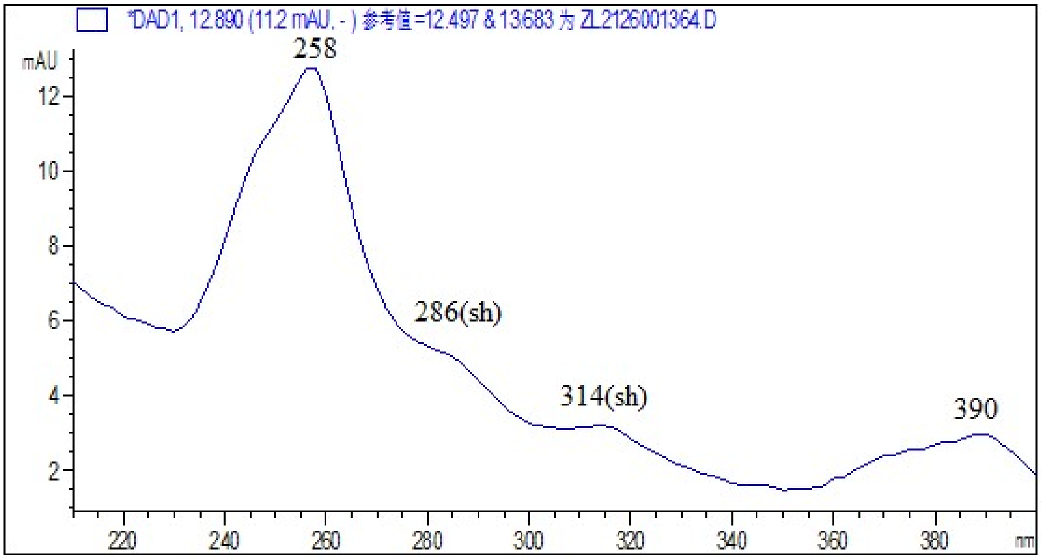

S3. IR spectrum of compound **9.**

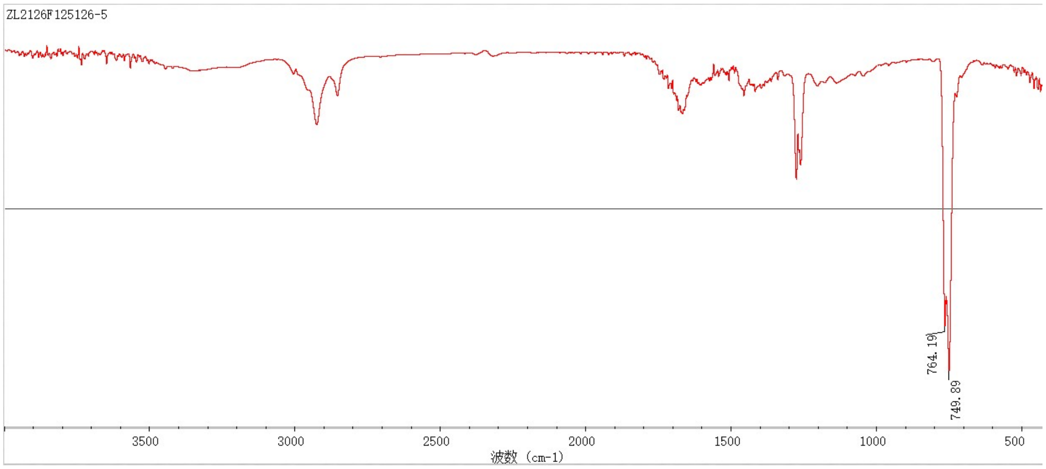

S4. ^{1}H NMR spectrum of compound **9** (DMSO-d_6, 500 MHz).

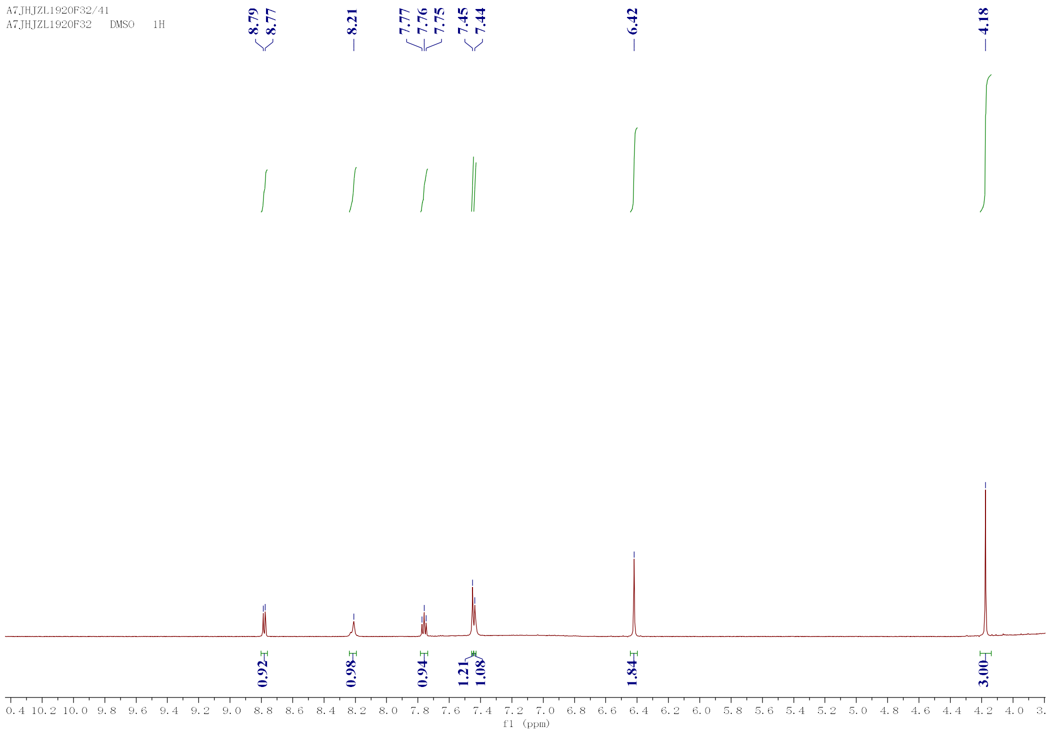

S5. ^{13}C NMR spectrum of compound **9** (DMSO-d_6, 125 MHz).

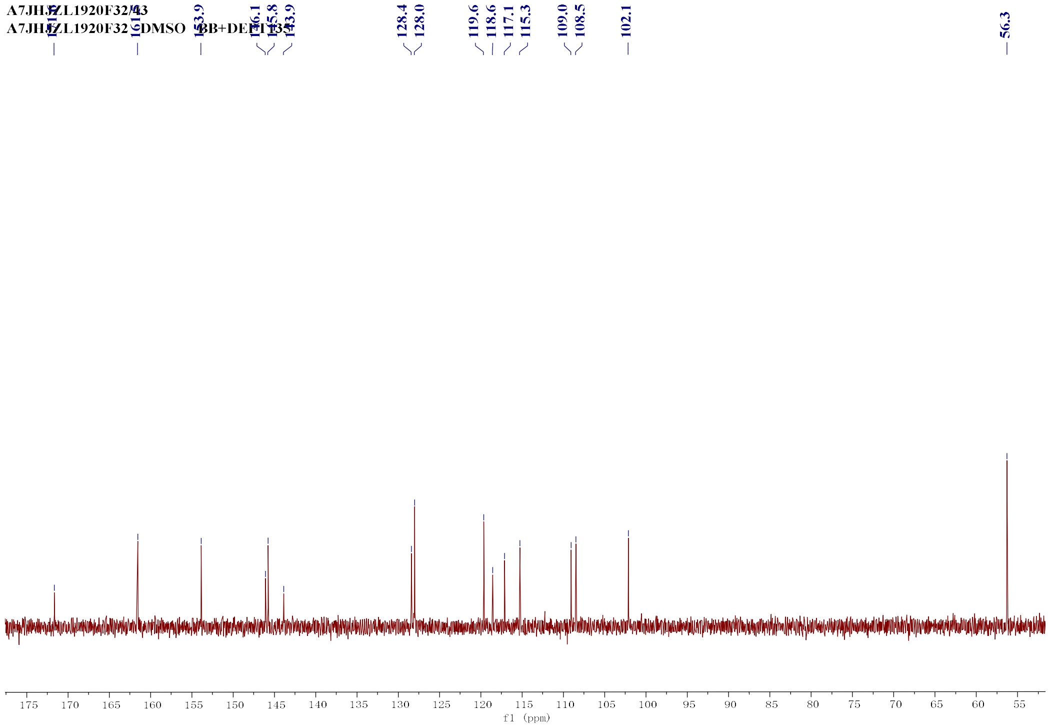

S6. HSQC spectrum of compound **9** (DMSO-d_6, 500 MHz).

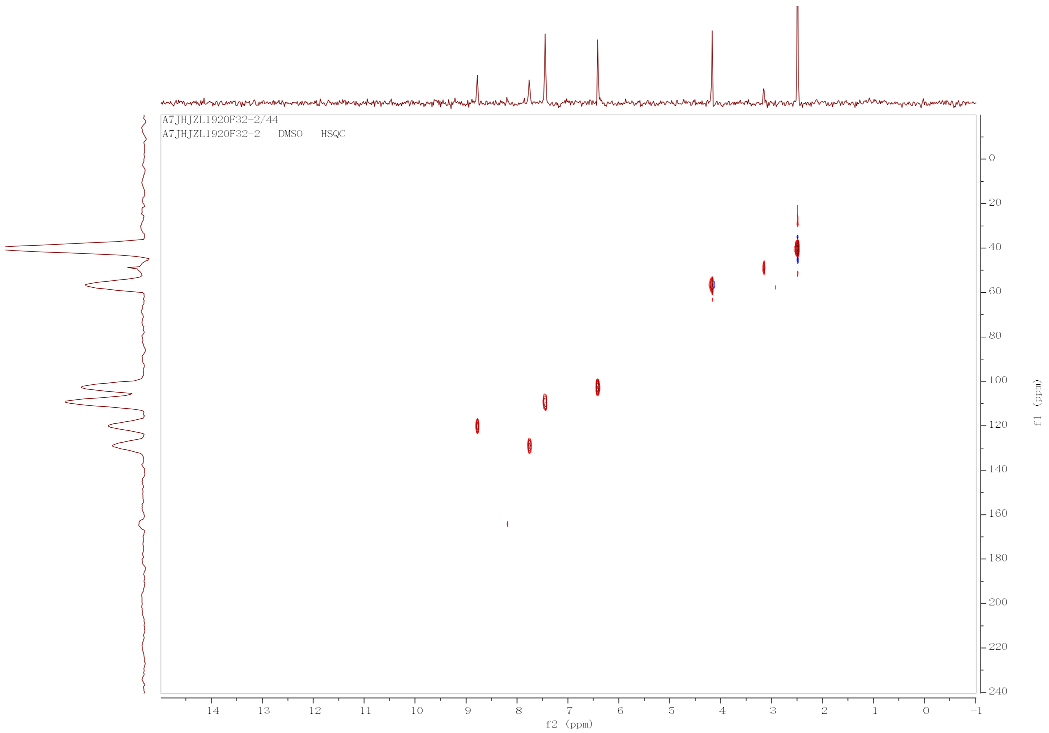

S7. HMBC spectrum of compound **9** (DMSO-d_6, 500 MHz).

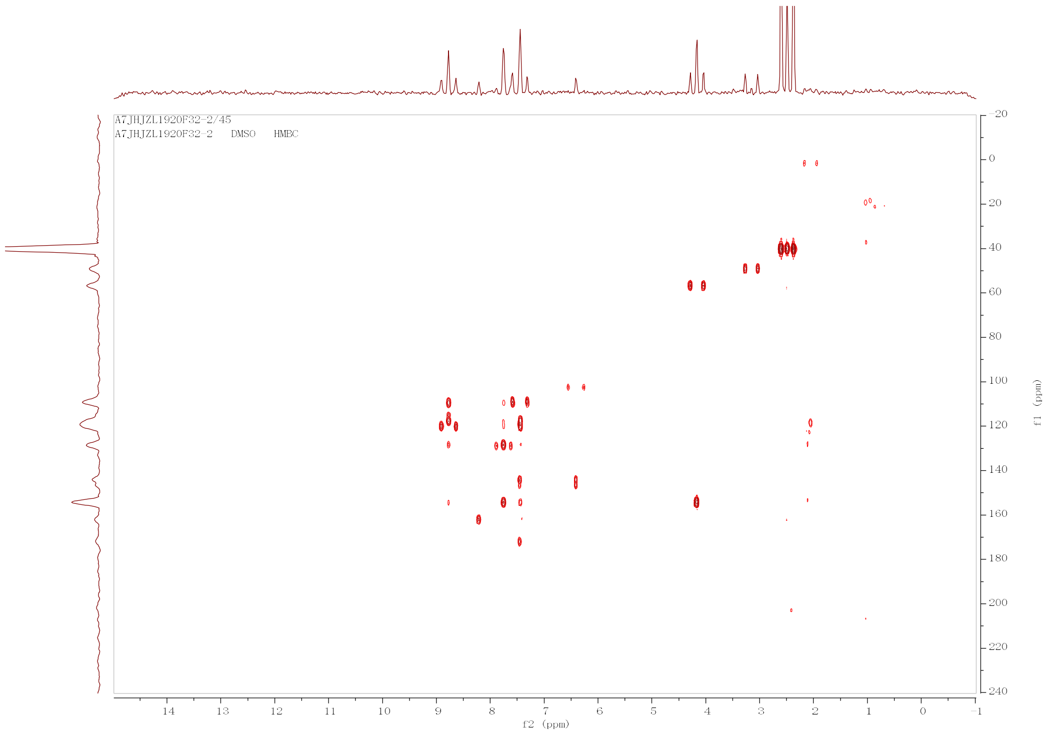

S8. A: tR of compound 9;

B: *m/z* of compound 9 at the same retention time;

C: TIC of the methanol extract from root of A.*contorta* by LC-Q-TOF/MS;

D: EIC of *m/z* 397.03 from the methanol extract from root of A.*contorta* by LC-Q-TOF/MS under the same liquid chromatography and mass spectrometry conditions;

E: Compound **9** was identified from the methanol extract from root of A. *contorta* by LC-Q-TOF/MS under the same liquid chromatography and mass spectrometry conditions. As indicated by symbols # and *.

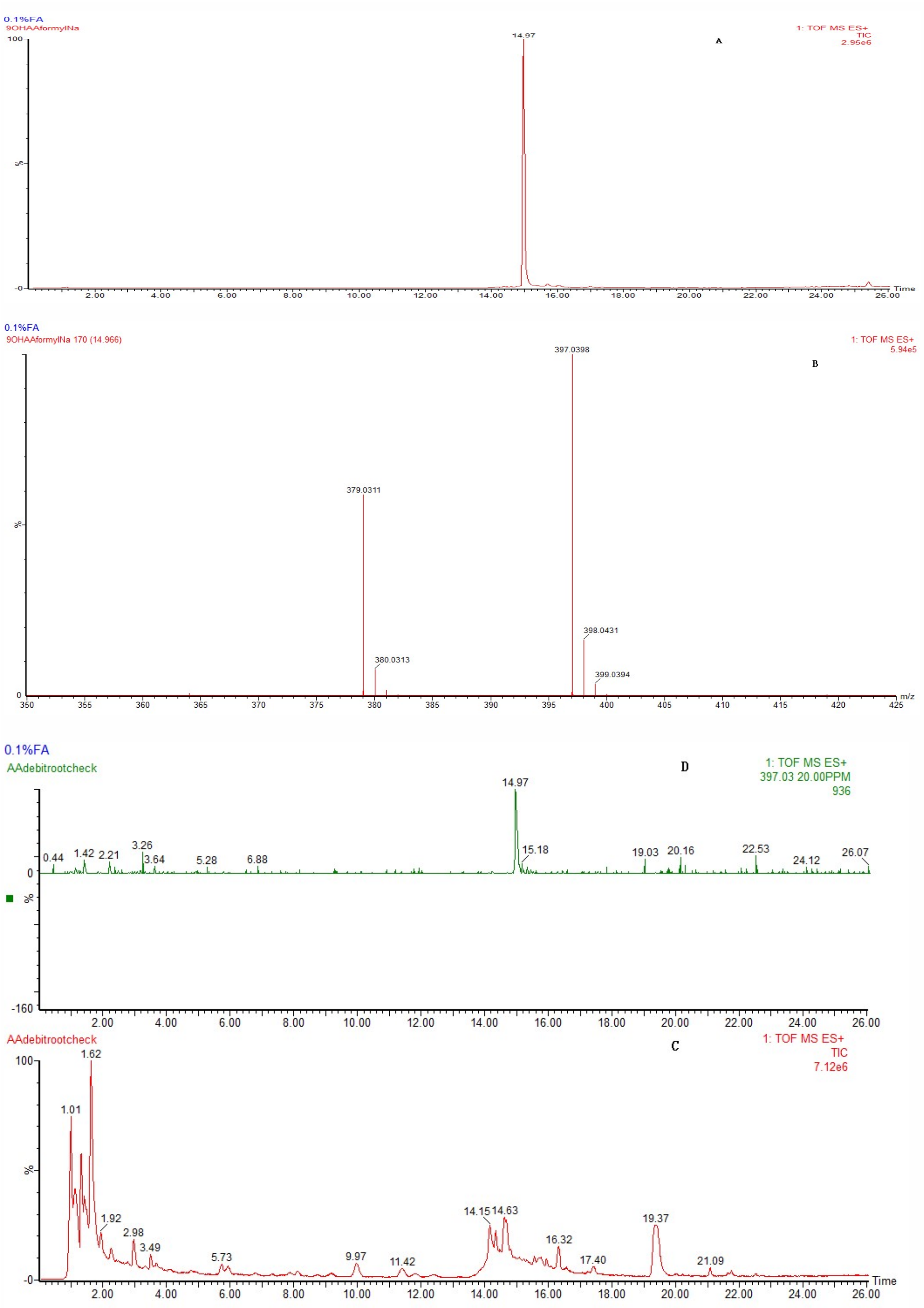
0.1%FA
9OHAAformylNa
A
1: TOF MS ES+
TIC
2.95e6
14.97
Time
0.1%FA
9OHAAformylNa 170 (14.966)
B
1: TOF MS ES+
5.94e5
397.0398
379.0311
398.0431
380.0313
399.0394
m/z
0.1%FA
AAdebitrootcheck
D
1: TOF MS ES+
397.03 20.00PPM
936
14.97
0.44
1.42
2.21
3.26
3.64
5.28
6.88
15.18
19.03
20.16
22.53
24.12
26.07
AAdebitrootcheck
C
1: TOF MS ES+
TIC
7.12e6
1.62
1.01
1.92
2.98
3.49
5.73
9.97
11.42
14.15
14.63
16.32
17.40
19.37
21.09
Time

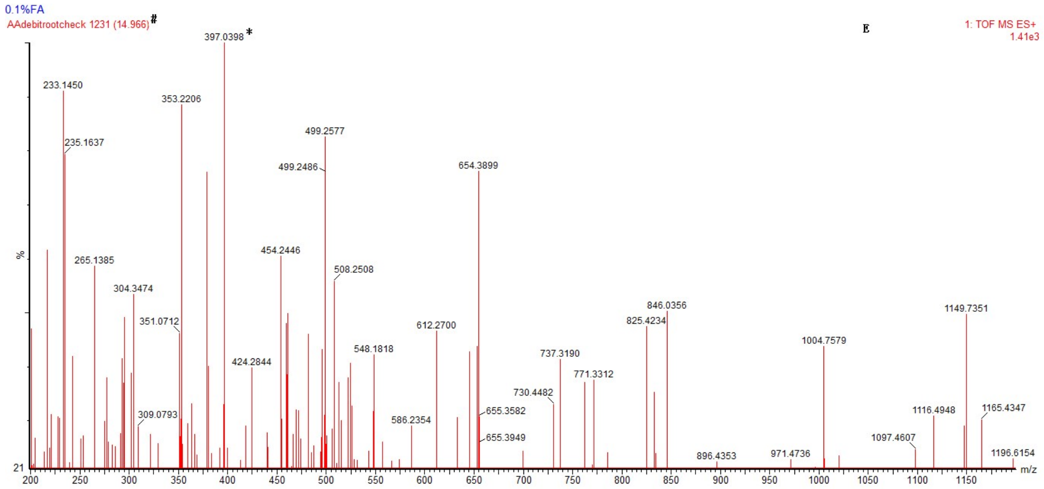

S9. HR-ESI–MS spectrum of compound **10.**

HR-ESI(+)–MS

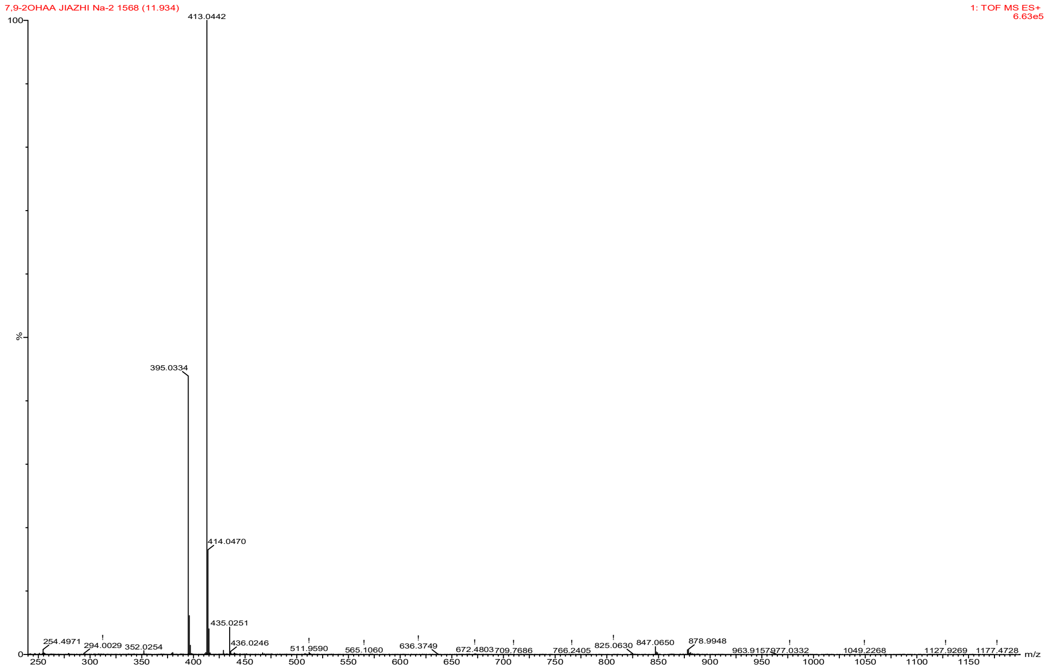

HR-ESI(−)–MS

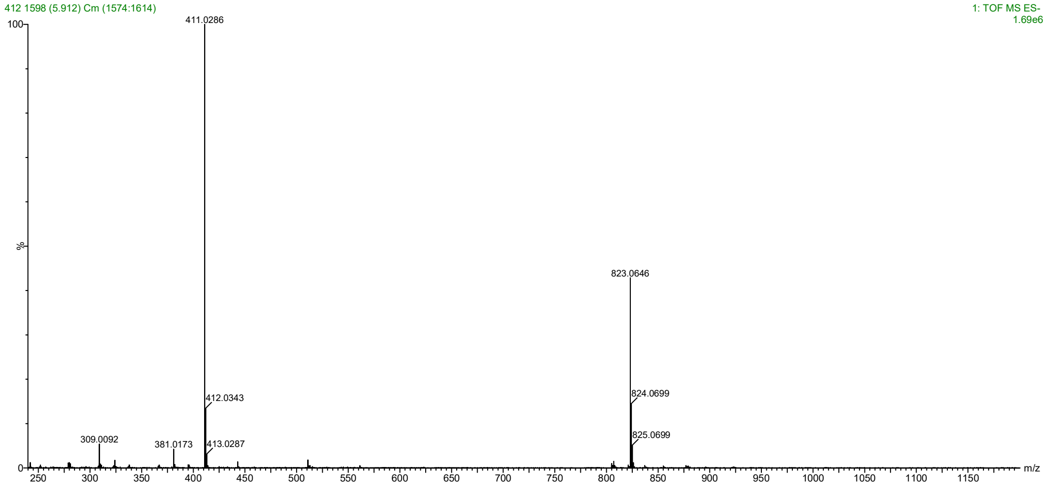

S10. UV spectrum of 10 (methanol).

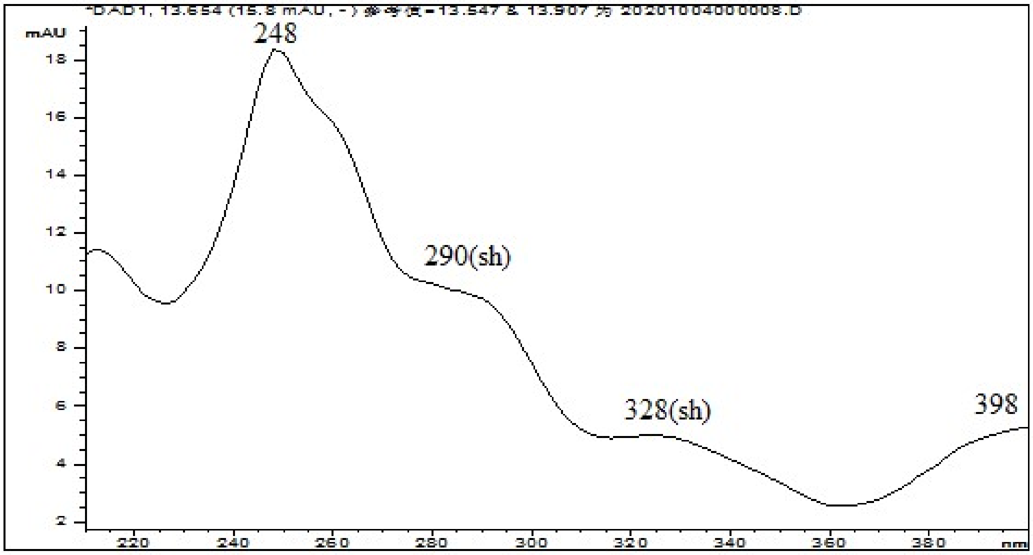

S11. 1H NMR spectrum of 10 (DMSO-*d6*, 500 MHz).

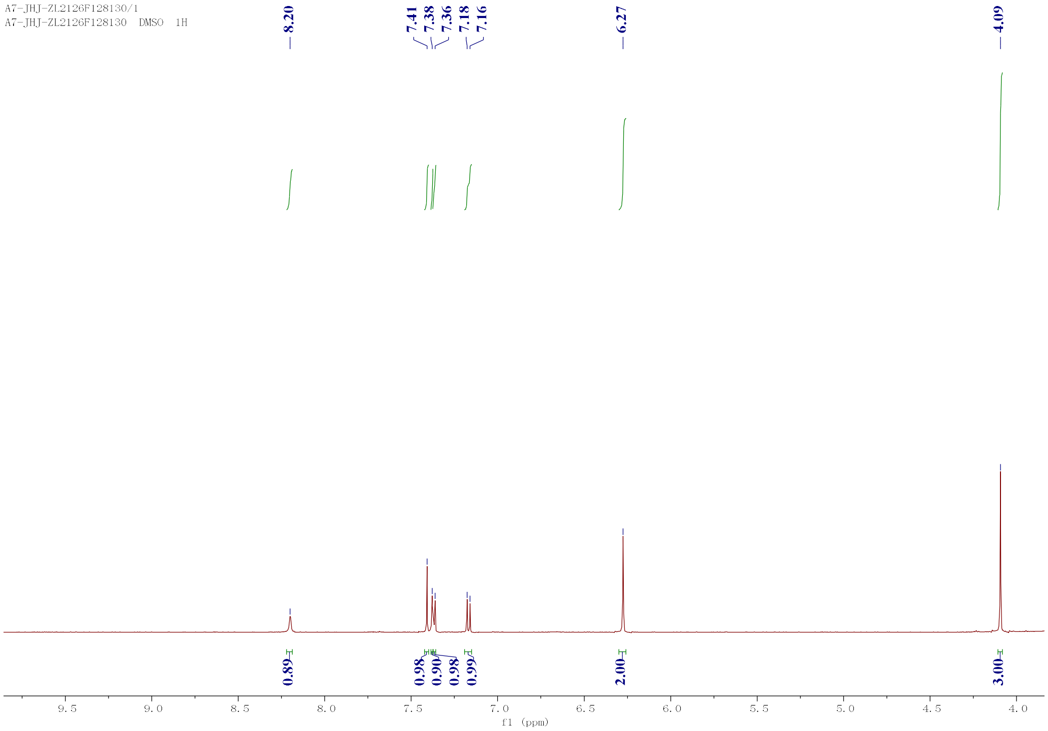

S12. The ^{13}C NMR spectrum of compound **10** (DMSO-d_6, 125 MHz).

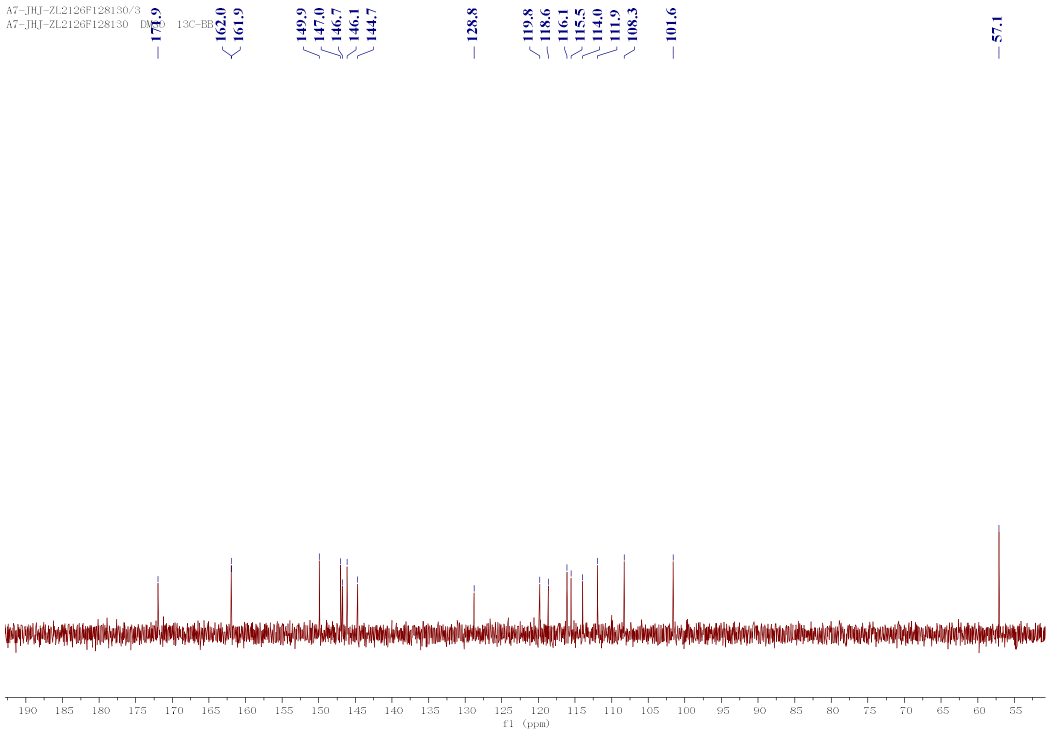

S13. HSQC spectrum of compound **10** (DMSO-d_6, 500 MHz).

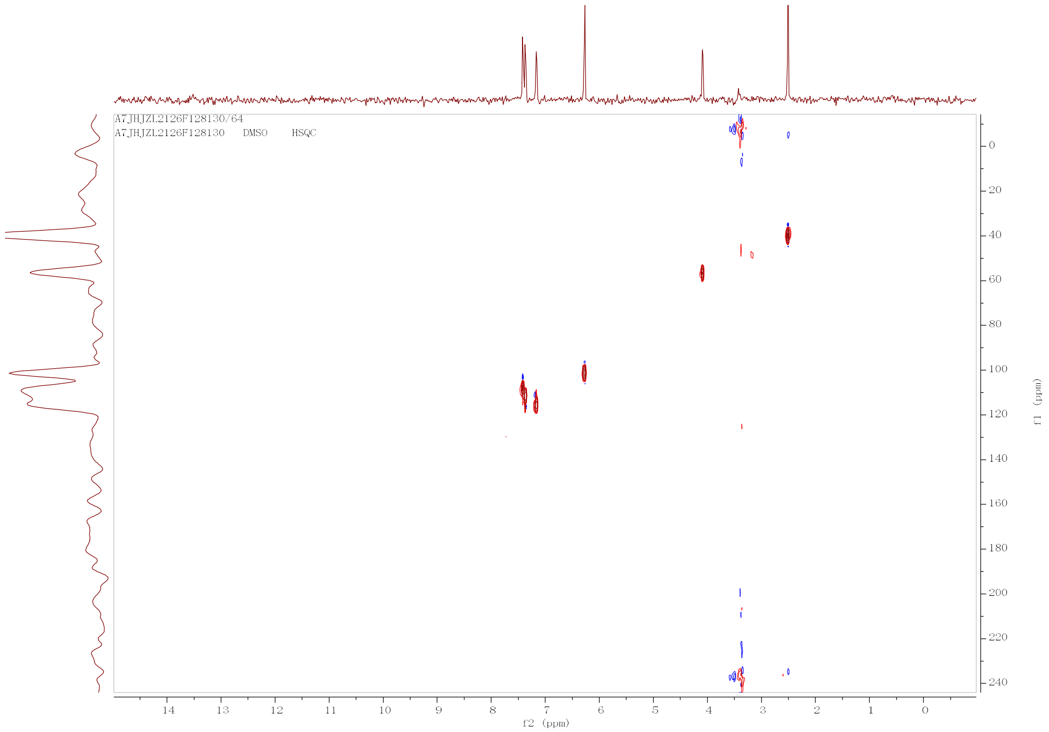

S14. F: tR of compound 10;

G: *m/z* of compound 10 at the same retention time;

H: TIC of the methanol extract from root of A. *contorta* by LC-Q-TOF/MS;

I: EIC of *m/z* 413.035 from the methanol extract from root of A.*contorta* by LC-Q-TOF/MS under the same liquid chromatography and mass spectrometry conditions;

J: Compound **10** was identified from the methanol extract from root of A.*contorta* by LC-Q-TOF/MS under the same liquid chromatography and mass spectrometry conditions. As indicated by symbols & and δ .

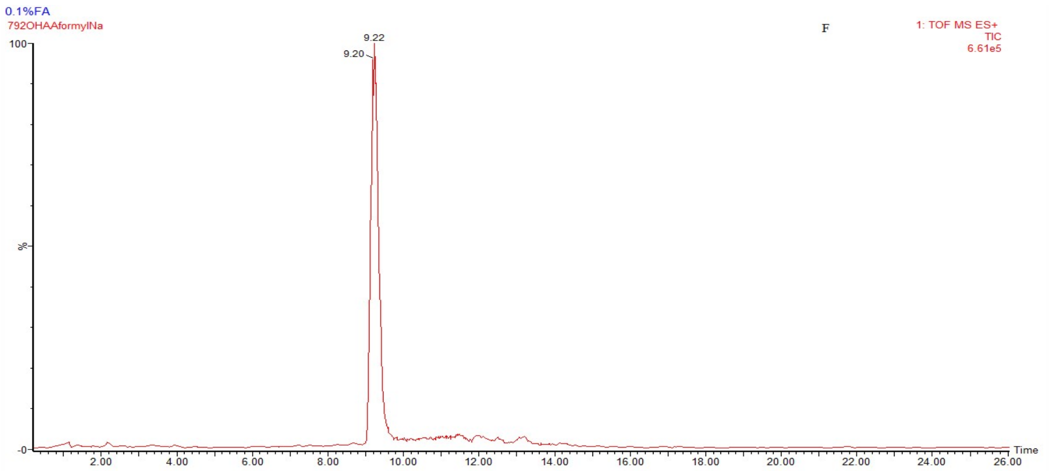

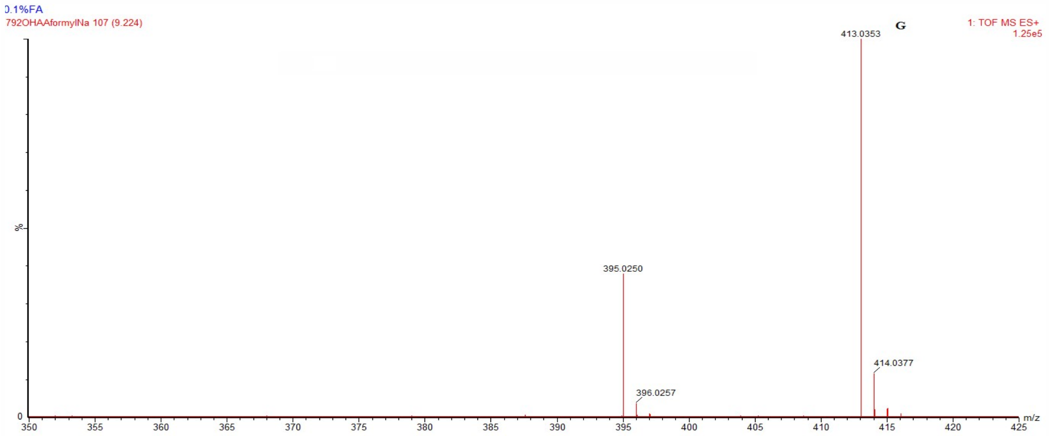

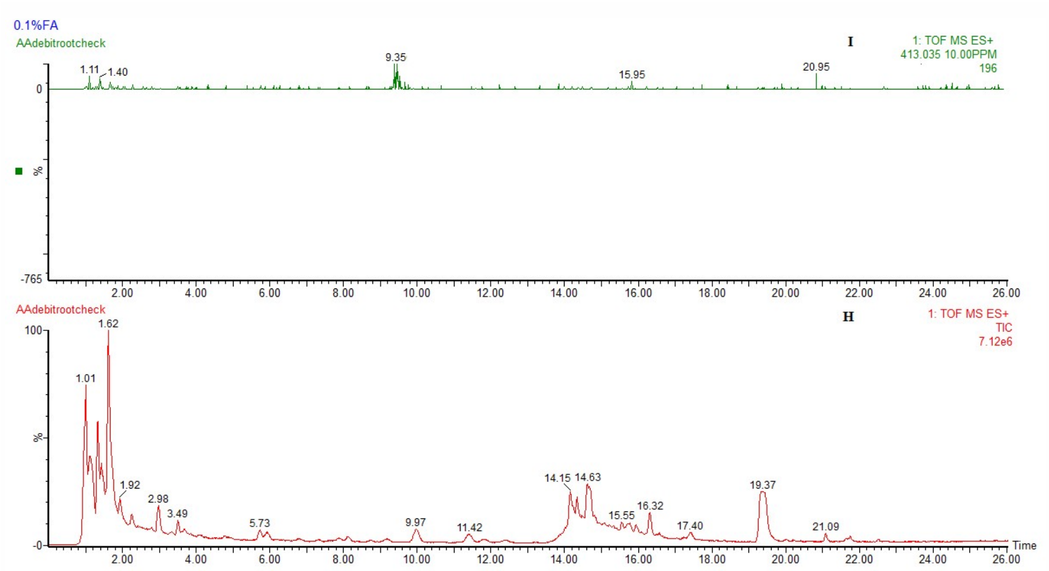

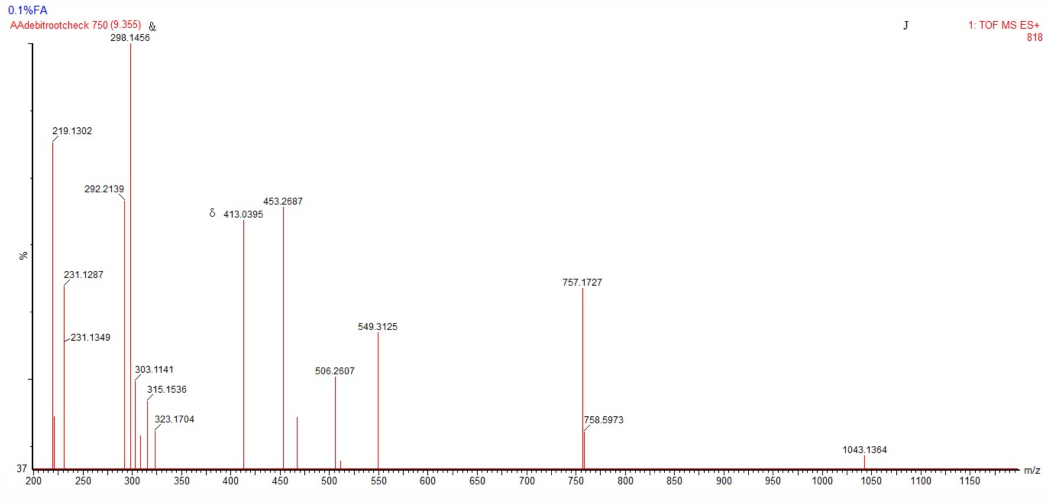

S15. ^{1}H NMR (DMSO-d_6, 500 MHz) and ^{13}C NMR (DMSO-d_6, 125 MHz) spectral data for compounds **1, 3, 5, 6,** and **7 (δ in ppm, *J* in Hz)**.

	1		3		5		6		7	
C	δ_H	δ_C	δ_H	δ_C	δH	δC	δH	δ_C	δ_H	δ_C
1		128.5		124.3		124.1		120.4		121.5
2	7.8, s	111.8	7.8, s	112.2	7.7, s	110.8	7.74, s	112.4	7.75, s	111.7
3		146		146		145.8		145.8		145.4
4		146.4		146.7		146.3		146.2		145.8
4a		117.2		116.8		117.8		117.1		117.3
4b		128.		129.		132.		132.		132.

		8		9		7		1		4
5	9.10, d (8.4)	126.5	8.65, d(8.5)	118.4	8.70, d(9.05)	121.9	8.09, s	103.1	8.48, d (2.2)	111.1
6	7.83, t(7.3)	130.4	7.85, t(8.2)	131.6	7.50, d(9.05)	122.8	10.86, br,s(–OH)	161.7		159.8
7	7.92, t(7.6)	128.7	7.37, d(8.1)	108.9	10.44, br,s(–OH)	148.4	6.84, s	104.2	7.29, dd (8.7, 2.2)	116.6
8	8.27, d(7.9)	130.4		156.3		148.9		158.5	8.09, d(8.7)	130.9
8a		129.6		118.8		122.8		112.6		121.5
9	8.58, s	125.8	8.58, s	119.4	8.40, (1H, s)	121.5	8.46, s	120.5	8.48, s	126.3
10		146		146		142.9		143.2		143.5
10a		116.6		117.3		115.5		118.2		118.7
-OCH3			4.07, s	56.3	3.98, s	60.9	4.01, s	56.6		
-CH2O-	6.51, s	102.9	6.50, s	102.9	6.45, s	102.6	6.46, s	100.2	6.49, s	102.7

S16.^{1}H NMR (DMSO-d_6, 500 MHz) and ^{13}C NMR (DMSO-d_6, 125 MHz) spectral data for compounds **2, 4, 8, 11,** and **12 (δ in ppm, *J* in Hz)**.

	2		4		8		11		12	
C	δ_H	δ_C	δ_H	δ_C	δ_H	δ_C	δ_H	δ_C	δ_H	δ_C
1		119.3		122.2		124.1		118.1		117.3
2	7.66,s	105.8	7.63,s	113.4	7.77,s	106.5	7.72,s	106	7.74,s	105.6
3		148.9		152.3		149.4		148.8		148.7
4		147.2	10.77,s	148.8		148.1		147.8		147.5
4a		110		120.3		111		111.2		111.3

4b		124.9		126.7		124.8		126		125.2
5	8.1,d(6.2)	118.8	9.11,d(7.7)	126	8.21,d(8.05)	118.2	8.01,d,(2.5)	118	8.08,*d*(8.1)	117.6
6	7.52, t(6.6)	125	7.52-7.58, m	125.2	7.60,t(8.05,8.05)	126.9		156.2	7.43,t(7.9,7.8)	126.4
7	7.21,d(6.6)	108.4	7.52-7.58, m	127.2	7.26,d(8.05)	111.6	7.13,dd(2.5,6.1)	118.2	7.12, *d*(7.8)	112.5
8		155.3	7.93,d(8.55)	128.9		155.9	7.87,d(8.7)	130.9		154
8a		124		134.8		119.2		126.9		122.8
9	7.36,s	97.9	7.09,s	103.8	7.66,s	109.1	7.35,s	107.9	7.64,s	101.1
10		132.8		135.3		134.1		131.9		132.7
10a		125.8		121.7		125.4		125.1		124.5
CONR	10.77,s	168.2		168.4		166.6		166.5		166.2
-OCH2O-	6.48,s	103.3			6.51,s	103.9	6.52,s	103.7	6.50,*s*	103.3
-OCH3	4.00,s	55.9	4.02,s	59.43	4.02,s	56.5				
1"					5.35,d(9.3)	82.8	5.34,d(9.5)	82.3	5.35,d(9.3)	81.8
2"						70.9		70.6		69.9
3"						77.9		78		77.5
4"						72.8		70.3		70.4
5"						80.6		80.6		80.2
6"						61.8		61.8		61.3

S17. Structure of PDB code **4NOG**.

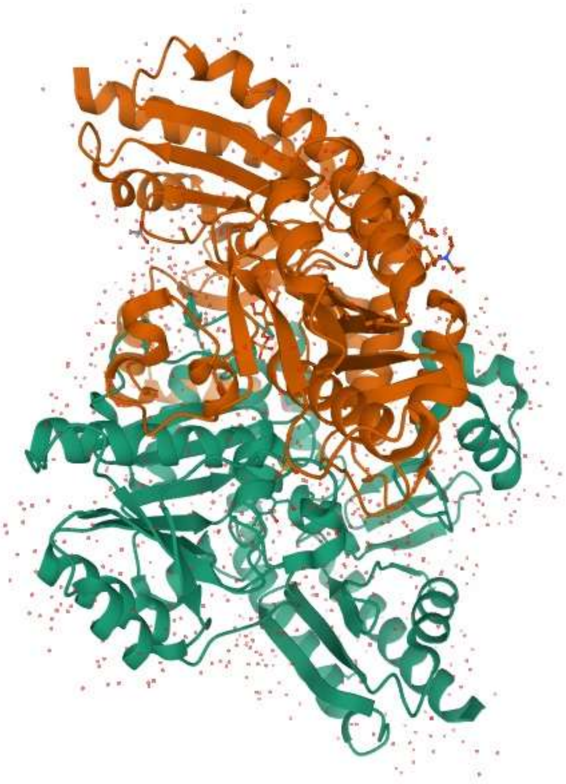

S18. **4NOG** generated with ligand by SWISS-MODEL software get 45.39% identity with OAT1.

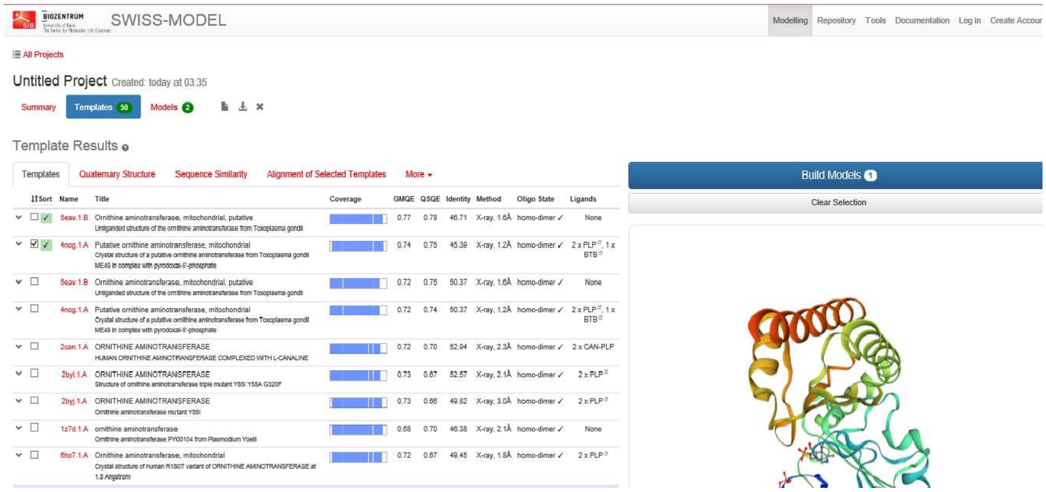

Name	Title	GMQE	QSQE	Identity	Method	Oligo State	Ligands
5eav.1.B	Ornithine aminotransferase, mitochondrial, putative<br>Unliganded structure of the ornithine aminotransferase from Toxoplasma gondii	0.77	0.78	46.71	X-ray, 1.6Å	homo-dimer ✓	None
4nog.1.A	Putative ornithine aminotransferase, mitochondrial<br>Crystal structure of a putative ornithine aminotransferase from Toxoplasma gondii ME49 in complex with pyrodoxal-5'-phosphate	0.74	0.75	45.39	X-ray, 1.2Å	homo-dimer ✓	2 x PLP, 1 x BTB
5eav.1.B	Ornithine aminotransferase, mitochondrial, putative<br>Unliganded structure of the ornithine aminotransferase from Toxoplasma gondii	0.72	0.75	50.37	X-ray, 1.6Å	homo-dimer ✓	None
4nog.1.A	Putative ornithine aminotransferase, mitochondrial<br>Crystal structure of a putative ornithine aminotransferase from Toxoplasma gondii ME49 in complex with pyrodoxal-5'-phosphate	0.72	0.74	50.37	X-ray, 1.2Å	homo-dimer ✓	2 x PLP, 1 x BTB
2can.1.A	ORNITHINE AMINOTRANSFERASE<br>HUMAN ORNITHINE AMINOTRANSFERASE COMPLEXED WITH L-CANALINE	0.72	0.70	52.94	X-ray, 2.3Å	homo-dimer ✓	2 x CAN-PLP
2byl.1.A	ORNITHINE AMINOTRANSFERASE<br>Structure of ornithine aminotransferase triple mutant Y85I Y55A G320F	0.73	0.67	52.57	X-ray, 2.1Å	homo-dimer ✓	2 x PLP
2byj.1.A	ORNITHINE AMINOTRANSFERASE<br>Ornithine aminotransferase mutant Y85I	0.73	0.66	49.82	X-ray, 3.0Å	homo-dimer ✓	2 x PLP
1z7d.1.A	ornithine aminotransferase<br>Ornithine aminotransferase PY00104 from Plasmodium Yoelii	0.68	0.70	46.38	X-ray, 2.1Å	homo-dimer ✓	None
6hx7.1.A	Ornithine aminotransferase, mitochondrial<br>Crystal structure of human R180T variant of ORNITHINE AMINOTRANSFERASE at 1.8 Angstrom	0.72	0.67	49.45	X-ray, 1.8Å	homo-dimer ✓	2 x PLP
